# The reliability and validity of rapid transcranial magnetic stimulation mapping for muscles under active contraction

**DOI:** 10.1186/s12868-024-00885-w

**Published:** 2024-08-30

**Authors:** Nahian S. Chowdhury, Wei-Ju Chang, Rocco Cavaleri, Alan K.I. Chiang, Siobhan M. Schabrun

**Affiliations:** 1 Center for Pain IMPACT, Neuroscience Research Australia, 139 Barker Street, Randwick, Sydney, NSW 2031 Australia; 2 University of New South Wales, Sydney, NSW Australia; 3 School of Health Sciences, College of Health, Medicine and Wellbeing, The University of Newcastle, Callaghan, NSW Australia; 4 Brain Stimulation and Rehabilitation (BrainStAR) Lab, School of Health Sciences, Western Sydney University, Sydney, NSW Australia; 5 The Gray Centre for Mobility and Activity, Parkwood Institute, London, Canada; 6 School of Physical Therapy, University of Western Ontario, London, Canada

**Keywords:** TMS mapping, Corticomotor excitability, Reliability, Validity, Neuroplasticity

## Abstract

Rapid mapping is a transcranial magnetic stimulation (TMS) mapping method which can significantly reduce data collection time compared to traditional approaches. However, its validity and reliability has only been established for upper-limb muscles during resting-state activity. Here, we determined the validity and reliability of rapid mapping for non-upper limb muscles that require active contraction during TMS: the masseter and quadriceps muscles. Eleven healthy participants attended two sessions, spaced two hours apart, each involving rapid and ‘traditional’ mapping of the masseter muscle and three quadriceps muscles (rectus femoris, vastus medialis, vastus lateralis). Map parameters included map volume, map area and centre of gravity (CoG) in the medial-lateral and anterior-posterior directions. Low to moderate measurement errors (%SEM_eas_ = 10–32) were observed across muscles. Relative reliability varied from good-to-excellent (ICC = 0.63–0.99) for map volume, poor-to-excellent (ICC = 0.11–0.86) for map area, and fair-to-excellent for CoG (ICC = 0.25–0.8) across muscles. There was Bayesian evidence of equivalence (BF’s > 3) in most map outcomes between rapid and traditional maps across all muscles, supporting the validity of the rapid mapping method. Overall, rapid TMS mapping produced similar estimates of map parameters to the traditional method, however the reliability results were mixed. As mapping of non-upper limb muscles is relatively challenging, rapid mapping is a promising substitute for traditional mapping, however further work is required to refine this method.

**Supplementary Information:**

The online version contains supplementary material available at 10.1186/s12868-024-00885-w.

## Highlights

We assessed the validity and reliability of rapid mapping for muscles that require active contraction during TMS: masseter and quadriceps.

Rapid TMS mapping showed equivalence in map parameters with the “gold standard”, however relative reliability was mixed.

Rapid TMS mapping is a promising substitute for traditional TMS mapping, but reliability of some parameters remains questionable.

**Supplementary Information:**

The online version contains supplementary material available at 10.1186/s12868-024-00885-w.

## Introduction

Transcranial magnetic stimulation (TMS) is a valuable tool for studying the structure and function of the primary motor cortex (M1) [1]. Using TMS, a “map” of the corticomotor representation of a target muscle can be obtained. These maps provide information about corticomotor excitability as well as the location of the corticomotor representation [2]. These measures are important for understanding neuroplasticity in healthy and clinical populations, including how corticomotor representations of muscles are affected by neurological disorders [3], motor task training [4; 5; 6] and pain [7; 8; 9; 10].

Traditionally, mapping is performed by delivering TMS pulses at multiple predefined sites, organised in ∼ 1 cm spaced rows and columns, with multiple stimuli delivered at each 1cm^2^ area [11; 12; 2]. However, the traditional mapping method can be lengthy because the experimenter must be spatially precise where TMS is delivered and cover the entirety of the surface representation. The recent development of neuronavigation software has allowed mapping to be as quick as two minutes using the “rapid mapping method” [13; 14]. In this method, TMS is delivered pseudorandomly within a specified area (e.g., a 6 × 6 cm^2^ area) rather than at predetermined grid sites. The motor evoked potential (MEP) amplitudes recorded at each stimulation location are then used to construct an estimated brain map via interpolation. This method has been shown to produce corticomotor maps that (a) have acceptable measurement error (absolute reliability), (b) have acceptable consistency between sessions (relative reliability), and (c) produce map parameter estimates similar to the traditional method (validity) in muscles of the upper limb and hand [15, 16, 13; 17; 18; 14].

Thus far, the rapid mapping technique has only been validated during resting muscle activity. However, certain muscles require active contraction to obtain reliable MEPs due to the depth of cortical representations, with two examples being the masseter and quadriceps muscles [11; 19]. Assessing the reliability and validity of rapid mapping for such muscles is vital for many areas of research. For example, mapping of the masseter and quadriceps has been useful for understanding the neurophysiology of chronic musculoskeletal pain conditions such as temporomandibular disorder [20] and patellofemoral pain [19], wherein alterations in motor representations of masseter and quadriceps muscles have been demonstrated respectively. Unfortunately, the time-consuming nature of traditional TMS mapping and the need to maintain muscle contraction can lead to muscle fatigue, potentially reducing data quality. Rapid TMS mapping can significantly reduce the acquisition time and overcome the shortcomings of the traditional method. However, whether corticomotor maps for the masseter and quadriceps obtained using the rapid method exhibit acceptable reliability and validity remains unknown.

Thus, this study aimed to determine the absolute reliability, relative reliability, and validity of corticomotor maps for the masseter and quadriceps muscles using rapid TMS mapping in healthy adults.

## Methods

### Participants

Eleven healthy participants (five females and six males, mean age:29 ± 8 years) were recruited using advertisements emailed throughout Neuroscience Research, Australia. Upon arrival, the participants completed a TMS safety screen [21]. Participants were excluded if they experienced any pain, were pregnant, had any major medical, neurological, or psychiatric conditions, reported a previous history of lower limb injuries or surgeries, or were taking psychoactive medication at the time of testing.

### Experimental protocol

**Fig. 1:**
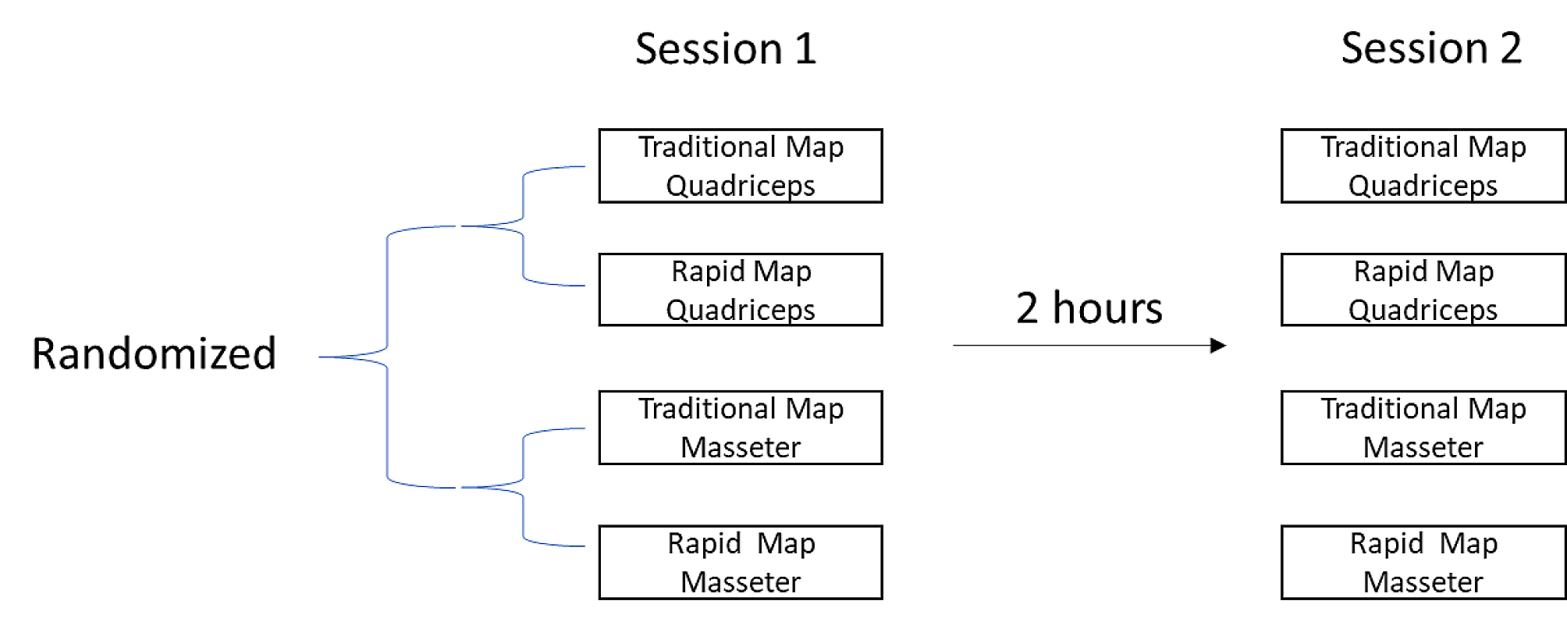
Experimental design

In line with a previous study, participants attended two sessions spaced two hours apart [13]. As we were interested in the reliability of the rapid mapping method itself, having a long intersession interval might lead to systematic changes in corticomotor excitability, making it difficult to disentangle whether differences between sessions are due to poor reliability or simply systematic changes in corticomotor excitability across time. For each session, maps were collected for the masseter and quadriceps muscles in a randomised order, using both traditional and rapid mapping methods (Fig. 1). One researcher (NC) conducted the masseter maps for all participants, while another (WJC) conducted the quadriceps maps for all participants. The researchers in this study are experienced in TMS mapping and have been involved in previous TMS masseter mapping and lower limb muscle mapping studies [20; 22].

### Electromyography

Participants were seated in a comfortable chair and viewed a monitor that displayed electromyographic (EMG) feedback. Bipolar surface electrodes were used to record motor evoked potentials (MEPs) from the right masseter muscle using a belly tendon montage with active (muscle belly) and reference (tendon) electrodes placed along the mandibular angle, and the ground electrode was placed on the right acromion process [20]. For corticomotor representations of the quadriceps muscles, bipolar surface electrodes were placed over the belly of the right rectus femoris (RF), vastus lateralis (VL), and vastus medialis oblique (VMO), with the ground electrode placed at the right tibial shaft. The electrode for measuring RF muscle activity was placed 15 cm proximal to the superior border of the patella on the reference line (a line drawn between the centre of the patella and the anterior superior ischial spine) [23]. The electrode for measuring VL activity was placed 15 cm proximal to the superior border of the patella in a line 20° lateral to the reference line [24]. The electrode for measuring VMO muscle activity was placed 45° medial to the reference line, 4 cm proximal to the superior patellar border, and 4 cm lateral to the reference line [24]. EMG signals were amplified (x2000) using a CED 1902, filtered (20–1000 Hz), and digitally sampled at 2000 Hz using a Power 1401 Data Acquisition System, and Spike2 software (CED Limited, Cambridge, UK). Participants were seated with the hips and knees at 90 degrees of flexion during the entire mapping procedure. Prior to the TMS setup, participants performed three 3s maximal muscle contractions for the masseter and RF muscles to calculate the average root mean square (RMS) EMG during maximum voluntary contraction (MVC). The contraction target for the subsequent hotspot, threshold and mapping procedures were 20% MVC for the masseter [20] and 10% MVC for the RF muscle [25]. To maintain 10% MVC of the RF muscle, participants were instructed to perform isometric knee extension against a theraband. The participants received live visual feedback from the EMG signals (which was rectified and smoothed for display purposes) to maintain their contraction within a range ± 2.5% of the target contraction level.

### TMS mapping procedures

While participants maintained the target contraction, the scalp sites evoking the largest MEP (the “hotspot”) for the masseter and RF muscles at a given TMS intensity were first determined. The active motor threshold (AMT) was determined using the TMS motor threshold assessment tool [26]. The AMT was defined as the minimum intensity required to evoke discernible MEPs. An MEP was identified if the EMG waveform was visibly larger in amplitude relative to the background EMG 5-15ms after the TMS pulse for the masseter [27; 28], and 26–46 ms for the RF muscle [29]. To minimise setup time, the hotspot locations and AMT values for the masseter and RF muscles in Session 1 were recorded and used in Session 2. Variations in these values within a two-hour intersession interval were expected to be negligible [5, 30]. The RF, VL, and VM were mapped concurrently in both sessions, as has been done previously [31].

Single monophasic stimuli were delivered using Magstim Rapid^2^ (Magstim Ltd., UK) and a 70 mm figure-of-eight coil at 120% AMT. An angle of 90° between the anterior-posterior line and medial-lateral line was used to induce a current in the lateral-to-medial direction [32; 33]. The Neural Navigator (Neurosoft, Russia) was used to track the positions of the TMS coil and the participant head in a 3-dimensional (3D) space. A model of the participant’s head was created by registering the position of anatomical landmarks (nasion, nose tip, and left and right ear tragus) into the software using a standard head-shape template.

TMS mapping was performed using a fixed 6 × 7 cm (7 rows and 8 columns) grid centred around the anticipated hotspot for the masseter (2 cm anterior and 6.5 cm lateral to the Cz) [34] and for the quadriceps muscles (0.5 cm anterior and 2 cm lateral to the Cz) [35]. For the traditional mapping method, three stimuli were delivered (interstimulus interval:4 s [13]) at each grid site (a total of 168 stimuli). For the rapid mapping method, 126 stimuli (interstimulus interval:2 s) were delivered using the pseudorandom walk method over the same grid as the traditional mapping. We based the number of stimuli from previous studies that used a stimulus density of 3 pulses per cm^2^ [13; 14]. This would also match the stimulus density of the traditional mapping method allowing for a fairer comparison. TMS stimuli were delivered in trains of three stimuli per grid site for the traditional mapping method and seven stimuli per train for the rapid mapping method. This was to provide breaks for participants in between contractions, thus minimizing muscle fatigue. As such, the interval between trains varied as the next train started when participants were ready for the next contraction. The neuronavigational display was monitored to ensure adequate coverage of the grid, and adjacent positions were not consecutively stimulated.

### Data processing

The MEP amplitude at each grid site was calculated as the average RMS EMG amplitude of MEP traces, subtracting the background RMS EMG 55 to 5ms prior to the TMS pulse [36; 37], using a custom MATLAB script (MATLAB 7, MathWorks Inc., USA). A fixed MEP onset/offset window was used (26-46ms post-TMS for the quadriceps muscles, 7-15ms post-TMS for the masseter muscle) based on MEP latencies from previous studies using TMS mapping in the quadriceps and masseter muscles [20, 29] and confirmed using onsets/offsets that were manually determined for each participant. This fixed MEP window method has been shown to yield highly consistent TMS map parameters when compared to manually determined MEP onsets/offsets for each trial [20].

**Fig. 2:**
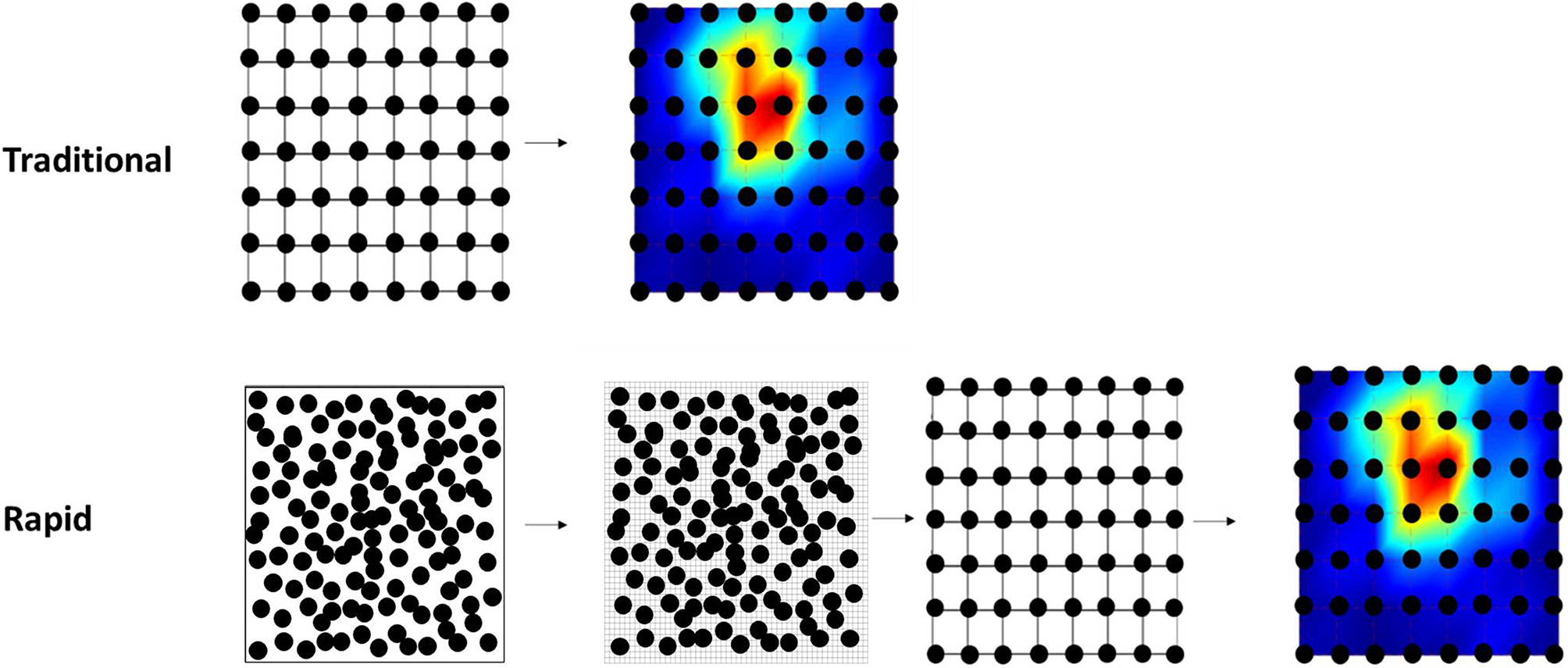
Schematic of map processing for the rapid and traditional mapping methods. For both methods, TMS was delivered over a 6 × 7 cm^2^ fixed area over the scalp. Traditional mapping consisted of three stimuli at 56 stimulation sites. The mean MEP amplitude at each site was calculated, and these values were used to create a map. The rapid mapping delivered 126 stimuli (3 stimuli/cm^2^). The 3D stimulation coordinates were transformed such that the 2D plane fitted through the coordinates was parallel to the transverse plane. This plane was then divided into 49 × 56 partitions, and MEP amplitude at each partition was interpolated using the nearest acquired MEP data. The mean MEP amplitude of each 7 × 8 sub-grid of partitions (representing the same locations as the traditional method) was then determined, and these values were used to create a map. Note the maps in this figure were generated from simulated data

Maps were processed using a custom MATLAB Script. For the traditional mapping method, MEP amplitudes (mean of each location) were superimposed over the respective grid sites to construct a topographical map. For the rapid mapping method, the stimulation coordinates were first transformed in 3D space so that a 2-dimension (2D) plane fitted through the stimulation coordinates was parallel to the transverse plane. The 2D plane was then divided into 2744 (49 × 56) partitions, and triangular linear interpolation was used to approximate the MEP amplitude based on the nearest acquired MEP data [14]. Because there were 56 stimulation locations for the traditional mapping method, the interpolated plane was then divided into 56 equally sized sub-grids representing the same locations that were stimulated for the traditional mapping method. The mean MEP amplitude of each sub-grid was calculated, and these values were used to construct a topographical map. Figure 2 shows a schematic representation of the two mapping methods.

The following map parameters were obtained for each map: (1) *map volume*, defined as the sum of all MEP amplitudes on the grid (interpolated grid in the case of rapid mapping) that were greater than 10% of the maximum MEP amplitude; (2) *map area*, defined as the number of sites (measured in cm^2^) on the grid which exhibited an MEP amplitude that was greater than 10% of the maximum MEP amplitude, and (3) *centre of gravity (CoG)*, defined as the amplitude-weighted centre of the map in either the anterior-posterior direction or the medial-lateral direction, using the following formula: (CoG=$\:\sum\:{V}_{i}x{\:X}_{i\:}/\sum\:{V}_{i}$, $\:\sum\:{V}_{i}x{\:Y}_{i\:}/\sum\:{V}_{i}$; *V*_*i*_=mean MEP amplitude at each site with the coordinates *X*_*i*_, *Y*_*i*_) [38, 12].

### Statistical analysis

Bland-Altman plots were used to assess homoscedasticity and the agreement between sessions and to visually inspect any systematic between-session differences and outliers for each map parameter of each target muscle [39; 40; 41; 42; 43]. When heteroscedasticity was identified, log transformation was performed and further analyses were conducted using log-transformed data.

#### Absolute reliability

Absolute Reliability was assessed using R, version 4.0.3 (R Development Core Team, Vienna, Austria) [44]. Absolute reliability, reflecting the measurement error and within-individual variability, was determined as the standard error of the measurement (SEMeas; a smaller SEMeas value indicates better measurement accuracy and lower within-individual variability) [39; 45; 42; 43]. The following formula was used: *SEMeas=√mean squared error*, where the mean squared error (or ‘residual error’) was obtained from one-way repeated measures analysis of variance applied to test and retest measurements [46]. SEMeas%, was also calculated (using the formula: SEMeas%= SEMeas/pooled mean*100%, where pooled mean was obtained from both testing sessions), to indicate the relative size of the measurement error [47; 42]. Additionally, the smallest detectable change (SDCi_ndiv_) at the individual level was derived from the SEMeas using the following formula: *SDC*_*indiv*_= SEMeas*1.96*√2 (for an individual), and minimal detectable change (MDC) for the group using the following formula: *SDC*_*group*_*= SDC*_*indiv*_*/√n; n = sample size* [48; 49; 42; 50]. Currently, there is no consensus on acceptable SDCs for TMS map parameters.

#### Relative reliability

The relative reliability was assessed using the MATLAB (Version 2021b) f_ICC function. To assess the relative reliability, the intraclass correlation coefficient (ICC) was evaluated using a single-rating, absolute-agreement, two-way mixed-effects model [ICC (2,1)] [39; 51; 43]. An ICC ≤ 0.2 indicates poor, 0.21 to 0.4 fair, 0.41 to 0.6 moderate, 0.61 to 0.8 good and > 0.8 excellent relative reliability [46].

#### Validity of rapid mapping method

To assess the validity of the rapid mapping method, we tested its equivalence against the traditional method [13] using equivalence Bayesian paired samples t-tests (paired-sample, equivalence interval: -0.25–0.25, non-overlapping hypotheses, Cauchy scale = 0.707) on JASP (Version 0.12.2.0, JASP Team, 2020) to compare the means of map volume, map area and CoG of the two sessions between the rapid and traditional mapping methods. A Bayes factor (BF) > 3 would provide at least moderate evidence that the rapid and traditional map parameters were inside the equivalence region, whereas a BF between 1 and 3 provided anecdotal/weak evidence that rapid and traditional map parameters were inside the equivalence region [52].

## Results

### Map characteristics

**Fig. 3:**
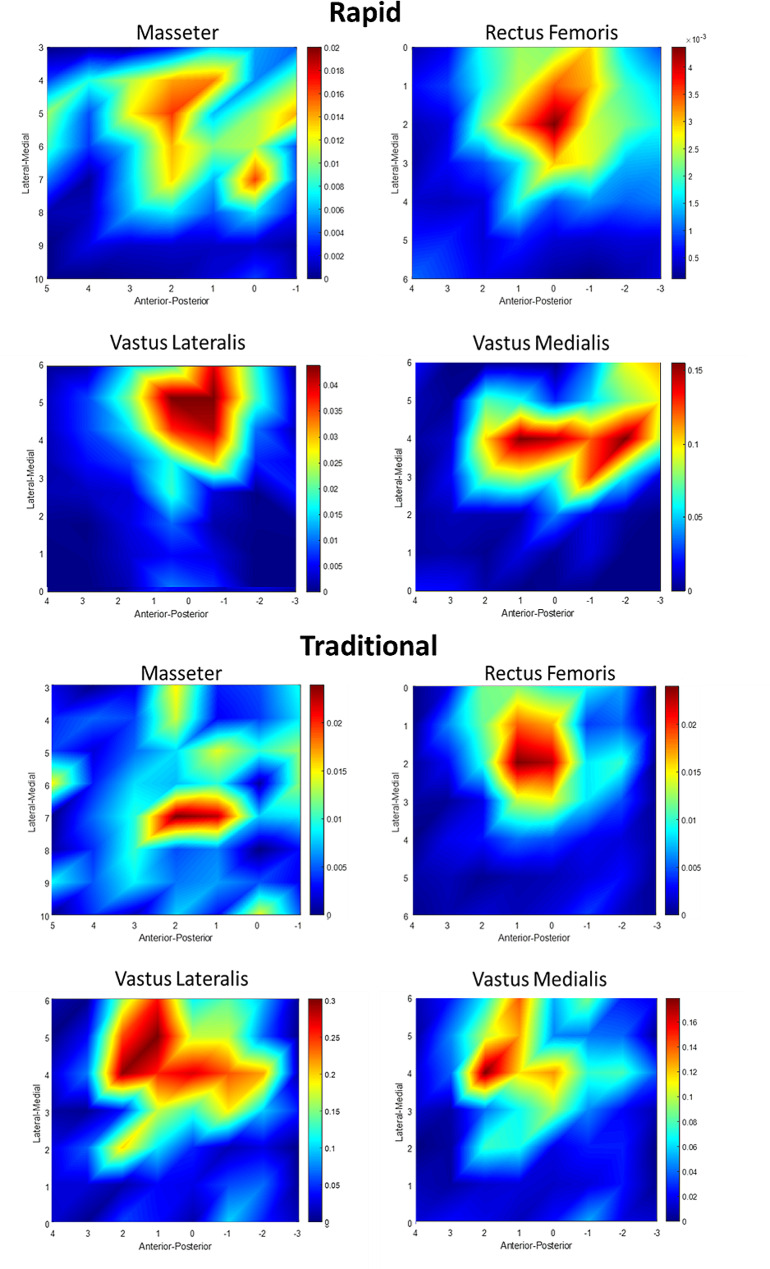
Map examples for each muscle obtained using the rapid and traditional mapping method. The axes represent the anterior-posterior and medial-lateral distance (in cm) relative to the vertex/Cz. Red and blue areas represent areas of higher and lower excitability respectively (measured in mV)

Owing to time constraints, one male participant did not complete the mapping protocols for the quadriceps muscles, and one female participant did not complete the mapping protocols for the masseter muscle. This led to ten participants (six males, four females) in the masseter muscle analysis, and ten participants (five females, five males) in the quadriceps muscle analysis. Table 1 shows the data for TMS procedural outcomes, including MVC, hotspot, AMT, and mapping times of both the rapid and traditional mapping methods. Rapid mapping was completed on average in ∼ 6–7 min, which was less than half the time required for traditional mapping (∼ 17 min). Table 2 lists the data for the map parameters of the rapid and traditional mapping methods for each muscle. Figure 3 shows examples of maps for each muscle from a representative participant obtained using the rapid and traditional mapping method.

**Table 1:** Measures relevant to rapid and traditional TMS mapping procedures for the masseter and rectus femoris muscles (mean, standard deviation and ranges)

		Session 1	Session 2
**Masseter**	MVC (mV)	0.05 ± 0.05 range: .02–.19	0.06 ± 0.03 range: .02–.15
	20%MVC (mV)	0.012 ± 0.01 range: .005–.04	0.018 ± 0.02 range: .005–.03
	Hotspot (cm, anterior to Cz)	2.6 ± 0.5 range: 2–3	–
	Hotspot (cm, medial to Cz)	6.8 ± 0.8 range: 5.5–7.5	–
	AMT (% MSO)	69.2 ± 12.7 range: 53–81	-
	120% AMT (% MSO)	83 ± 15.1 range: 64–97	–
	Rapid mapping time (mins)	6.9 ± 1.0 range: 6–9	6.1 ± 0.7 range: 6–7
	Traditional mapping time (mins)	16.6 ± 2.5 range: 14–22	17.3 ± 3.8 range: 14–26
**Rectus femoris**	MVC (mV)	0.03 ± 0.06 range: .004–.2	0.04 ± 0.07 range: .003–.19
	10%MVC (mV)	0.003 ± 0.006 range: .0004–.02	0.004 ± 0.007 range: .0003–.019
	Hotspot (cm, anterior to Cz)	1.1 ± 0.88 range: 0–2	–
	Hotspot (cm, medial to Cz)	2.1 ± 0.88 range: 0.5–3.5	-
	AMT (% MSO)	78.3 ± 7.98 range: 61–90	-
	120% AMT (% MSO)	93.7 ± 9.40 range: 73–108	–
	Rapid mapping time (mins)	6.5 ± 0.8 range: 5.5–8	7.0 ± 1.0 range: 6–8.75
	Traditional mapping time (mins)	17.8 ± 1.4 range: 16–20	16.5 ± 1.4 range: 15–19

*Note: AMT*,* active motor threshold; MSO = maximum stimulator output; MVC*,* maximum voluntary contraction*

**Table 2:** TMS map parameters (means and standard deviation) for the masseter and quadriceps muscles acquired by the rapid and traditional TMS mapping procedure

		Rapid		Traditional	
		Session 1	Session 2	Session 1	Session 2
**Masseter**	Map volume (mV)	0.80 ± 0.60	0.86 ± 0.75	0.85 ± 0.73	0.83 ± 0.57
	Map area (cm^2^)	39.30 ± 3.80	39.00 ± 6.20	40.30 ± 7.13	43.2 ± 7.51
	CoG X (cm, lateral to Cz)	6.20 ± 0.85	5.96 ± 0.85	6.09 ± 0.92	6.18 ± 0.75
	CoG Y(cm, anterior to Cz)	2.46 ± 0.87	2.73 ± 0.79	3.14 ± 0.8	3.25 ± 0.71
**Rectus femoris**	Map volume (mV)	0.34 ± 0.80	0.34 ± 0.82	0.31 ± 0.62	0.30 ± 0.67
	Map area (cm^2^)	30.4 ± 13.53	30.8 ± 10.97	31.60 ± 13.47	29.90 ± 11.03
	CoG X (cm, lateral to Cz)	2.20 ± 0.67	2.31 ± 0.62	2.18 ± 0.45	2.32 ± 0.58
	CoG Y (cm, anterior to Cz)	0.82 ± 0.73	1.03 ± 0.68	1.00 ± 0.72	1.18 ± 0.66
**Vastus lateralis**	Map volume(mV)	0.81 ± 0.60	0.9 ± 0.63	0.91 ± 0.74	0.68 ± 0.47
	Map area (cm^2^)	28.9 ± 7.49	29.7 ± 9.52	28.5 ± 5.42	28.4 ± 8.38
	CoG X (cm, lateral to Cz)	2.30 ± 0.69	2.47 ± 0.83	2.22 ± 0.49	2.42 ± 0.64
	CoG Y (cm, anterior to Cz)	1.03 ± 0.68	1.00 ± 0.85	0.82 ± 0.67	1.00 ± 0.56
**Vastus medialis**	Map volume (mV)	0.57 ± 0.50	0.68 ± 0.77	0.61 ± 0.48	0.58 ± 0.61
	Map area (cm^2^)	24.5 ± 7.55	28.5 ± 6.67	23.8 ± 7.50	23.8 ± 6.32
	CoG X (cm, lateral to Cz)	2.33 ± 0.68	2.53 ± 0.72	2.15 ± 0.55	2.38 ± 0.67
	CoG Y(cm, anterior to Cz)	0.83 ± 0.55	1.05 ± 0.87	0.78 ± 0.85	1.2 ± 0.77

*Note: CoG*,* centre of gravity*

### Reliability and SDC_indv_ of rapid TMS mapping for the masseter and quadriceps muscles

The results for the measurement error and the minimal detectable change at the individual level (SDC_indv_) are presented in Table 3. Measurement errors for rapid mapping were low for the map area (%SEM_eas_=10%) but moderate for the map volume and the CoG location coordinates (%SEM_eas_=10–32%) for the masseter and quadriceps muscles, with the exception of the map volume for the RF muscle (%SEM_eas_=10%). The results for relative reliability are presented in Table 4. For map volume, the relative reliability of the rapid mapping procedure ranged from good to excellent for the quadriceps and excellent for the masseter muscles. For map area, relative reliability of rapid mapping was poor for the masseter, and ranged from moderate-excellent for the quadriceps muscles. For CoG, the relative reliability for rapid mapping ranged from fair to moderate for the quadriceps muscles and good to excellent for the masseter.

**Table 3:** Absolute reliability results for the rapid mapping procedure

		Rapid			Traditional		
		SEM_eas_	%SEM_eas_	SDC_indv_	SEM_eas_	%SEM_eas_	SDC_indv_
**Masseter**	Map volume	0.61	32	1.7	0.56	23	1.5
	Map area	1.86	7	5.1	2.28	8	6.3
	CoG X	0.78	13	2.2	0.77	13	2.1
	CoG Y	0.77	21	2.1	0.73	18	2.0
**Rectus femoris**	Map volume	0.21	10	0.59	0.56	23	1.5
	Map area	2.55	10	7.1	2.44	11	6.8
	CoG X	0.67	20	1.9	0.77	13	2.1
	CoG Y	0.71	29	2.0	0.73	18	2.0
**Vastus lateralis**	Map volume	0.66	27	1.8	0.56	23	1.5
	Map area	2.46	10	6.8	2.23	11	6.2
	CoG X	0.73	21	2.0	0.77	13	2.1
	CoG Y	0.74	29	2.0	0.73	18	2.0
**Vastus medialis**	Map volume	0.57	26	1.6	0.56	23	1.5
	Map area	2.24	10	6.2	2.11	12	5.8
	CoG X	0.73	21	2.0	0.77	13	2.1
	CoG Y	0.71	29	2.0	0.73	18	2.0

*Note: CoG*,* centre of gravity; SDC*_*indv*_, *smallest detectable change; SEM*_*eas*_, *standard error of measurement*

**Table 4:** Intraclass correlation coefficient (ICC) data (and confidence intervals) for TMS map parameters of the masseter and quadriceps muscles acquired by the rapid mapping procedure

		Rapid	Traditional
**Masseter**	Map volume	0.85 (0.51–0.96)	0.96 (0.85–0.99)
	Map area	0.11 (-0.63, 0.68)	0.73 (0.24–0.92)
	CoG X	0.66 (0.09–0.90)	0.85 (0.50–0.96)
	CoG Y	0.85 (0.52–0.96)	0.86 (0.55–0.96)
**Rectus femoris**	Map volume	0.99 (0.99-1)	0.99 (0.98-1)
	Map area	0.86 (0.54, 0.96)	0.77 (0.31–0.94)
	CoG X	0.3 (-0.37-0.76)	0.47 (-0.19-0.83)
	CoG Y	0.31 (-0.35-0.77)	0.77 (0.33–0.94)
**Vastus lateralis**	Map volume	0.63 (0.04–0.9)	0.71 (0.23–0.9)
	Map area	0.43 (-0.23, 0.82)	0.70 (0.13–0.92)
	CoG X	0.20 (-0.43-0.71)	0.73 (0.28–0.93)
	CoG Y	0.67 (0.15–0.90)	0.68 (0.16–0.91)
**Vastus medialis**	Map volume	0.81 (0.42–0.95)	0.77 (0.29–0.94)
	Map area	0.49 (-0.16, 0.84)	0.50 (-0.21-0.85)
	CoG X	0.20 (-0.44-0.71)	0.85 (0.5–0.96)
	CoG Y	0.26 (-0.38-0.74)	0.74 (0.2–0.93)

*Note: CoG*,* centre of gravity*

### Validity of rapid TMS mapping for the masseter and quadriceps muscles

Table 5 presents the results of the Bayes factors for the equivalence tests between the rapid and traditional map parameters. There was moderate evidence of equivalence over non-equivalence between rapid and traditional mapping methods across all muscles (all BF’s > 3) with the exception of map area for the masseter, CoG Y for the masseter muscle and CoG X for the vastus medialis.

**Table 5:** Bayes factors (BF) from equivalence tests between the map parameters of the rapid and traditional mapping methods. The bold font represents substantial evidence for equivalence over non-equivalence (BF > 3)

		Bayes Factor
**Masseter**	Map volume	**3.6**
	Map area	2.97
	CoG X	**3.55**
	CoG Y	1.03
**Rectus femoris**	Map volume	**3.58**
	Map area	**6.33**
	CoG X	**3.60**
	CoG Y	**3.05**
**Vastus lateralis**	Map volume	**3.51**
	Map area	**5.91**
	CoG X	**3.50**
	CoG Y	**3.38**
**Vastus medialis**	Map volume	**3.58**
	Map area	**3.13**
	CoG X	2.94
	CoG Y	**3.55**

*Note: CoG*,* centre of gravity*

## Discussion

This study assessed the absolute reliability, relative reliability, and validity of the rapid TMS mapping method for the masseter and quadriceps muscles in healthy adults. The results showed low measurement error for map area, and mostly moderate measurement error for map volume and CoG. The relative reliability varied from good-to-excellent for map volume, poor-to-excellent for map area and fair-to-excellent for CoG coordinates. There was moderate Bayesian evidence of equivalence in the map parameters between the rapid and traditional maps in all muscles, supporting the validity of the rapid mapping method. Taken together, our findings provide reference values for minimal detectable changes in the masseter and quadriceps muscles in this population. While our results suggest the rapid mapping method produces similar estimates of map parameters to the traditional method, the reliability results of this method were mixed.

### Absolute reliability of rapid TMS mapping

As there are no accepted norms for measurement error (SEMeas) or SDC, an arbitrary cut-off value of < 10% for SEMeas% has been proposed to reflect low measurement error [53; 42]. Our results showed that rapid mapping of the masseter and quadriceps muscles had moderate measurement errors for map volume and CoG location coordinates (%SEM_eas_=10–32%), except for map volume of the RF muscle (SEMeas%=10%). Consistent with previous reports on SDCs of TMS mapping measures [18; 54] and other TMS measures [42], TMS map measures for the masseter and quadriceps muscles had sizable SDC_indv_, precluding evaluative use for tracking changes within an individual. Nevertheless, our data will enable future studies with similar cohort characteristics to estimate how much an individual (SDC_indv_) or group (SDC_indv_ divided by the square root of their sample size) would have to change in TMS map measures to be considered a real change exceeding the measurement errors [55]. As SDCs of TMS map measures for the masseter and quadriceps muscles become sufficiently low in modest sample sizes, they can be used for evaluative purposes to detect changes at the group level [42].

Research assessing the reliability of TMS mapping that requires muscle contractions during the procedure is scarce [56] and no study has examined the measurement error (absolute reliability). Higher TMS intensities are needed for mapping the facial and lower limb muscles at rest because of the depth of the M1 representation and the strength of corticospinal connections, which may not be tolerated by participants [56]. Thus, muscle contraction is necessary to allow mapping of these muscles with lower TMS intensities and better procedure tolerability [20; 29; 57]. The moderate measurement error of the TMS map for the masseter and quadriceps muscles may be attributed to between-session variation in the level of muscle contraction. Although 10% of MVC was set for the RF and 20% for the masseter muscles tailored to each participant and visual feedback of EMG was provided to maintain the target contraction during mapping, between-session variations in MVC within individuals may have influenced corticomotor representations of the mapped muscles.

### Relative reliability of rapid TMS mapping

Our results suggest that rapid mapping yields good-to-excellent relative reliability for the estimation of map volume. This aligns with previous studies demonstrating the excellent relative reliability of rapid mapping of the upper limb muscles [15, 16, 13]. Relative reliability results for the other outcomes were mixed: the quadriceps muscles showed moderate to excellent relative reliability for the map area but fair relative reliability for CoG, while the masseter muscle showed good to excellent relative reliability for CoG but poor relative reliability for the map area. These findings are contrary to those from other studies demonstrating excellent relative reliability for the map area and CoG estimates for the rapid mapping of upper limb muscles [17; 14]. One explanation for this could be the low number of stimuli required for rapid mapping of the quadriceps and masseter muscles. In this study, we chose 126 stimuli to match the three pulses per cm^2^ in traditional mapping; however, achieving equally distributed pulses across the grid space using the pseudorandom walk method is not always possible. Non-upper limb muscles have corticomotor representations that differ from other muscles, for example, a different number of discrete peaks of corticomotor excitability [58; 19]. It is conceivable that a higher number of pulses would increase the researcher’s ability to more evenly distribute TMS pulses within the grid. This may produce a more reliable interpolation of the map and improve the identification of active sites, thus producing more accurate estimates of the map area and CoG location coordinates [56]. This may be further aided by the use of robotic neuronavigated TMS maps [59] to ensure that the grid space contains a sufficient number and distribution of TMS pulses. In any case, despite the unexpected results for the map area and the CoG coordinates, rapid TMS mapping appears to produce stable measures of map volume for both masseter and knee muscles.

### Validity of rapid TMS mapping

Another aim of our study was to determine whether rapid mapping produces similar map estimates to the “gold-standard” traditional approach [12] where stimuli are delivered at 1 cm spaced points around the grid space. We used the same gold standard as Cavaleri et al. [13] where stimuli were delivered with a 4 s interstimulus interval. Our results showed that map volume, map area and most CoG assessments of the four muscles were equivalent between the rapid and traditional mapping methods. Although three comparisons yielded insufficient evidence, two of these (Map Area for Masseter, CoG X for VM) showed close to moderate evidence of equivalence (BF = 2.94–2.97). Overall, this suggests that while some map estimates obtained using the rapid mapping approach yield questionable relative reliability, they nonetheless produce similar map estimates to the “gold standard”. These results are mostly consistent with previous studies that showed equivalence between rapid and traditional maps [13; 14]. However, one previous study compared rapid and traditional mapping using null hypothesis testing [14], which does not appropriately assess equivalence, whereas the other used a frequentist approach for assessing equivalence [13], which requires corrections for multiple comparisons. A major strength of our study is the use of a Bayesian approach to assess equivalence. This approach allows researchers to quantify the strength of evidence for equivalence (rather than the accept/reject null hypothesis approach) [52] and does not require significance correction for multiple comparisons [60]. In summary, our findings support the validity of the rapid method and can be used instead of the traditional method.

### Limitations and future research suggestions

This study has several limitations. Although our sample size of 10 is similar to a previous reliability study on the rapid mapping method [14], this could have been optimized using a sample size calculation. In situations where sample size is not calculated, it has been recommended to provide confidence intervals or posterior Bayesian distributions [61] – the former is shown in Table 4 and latter has been added to the supplementary file. Another limitation is the lack of trial exclusion based on coil orientation/angle, as these data were not available from our neuronavigation system. Trials with inaccurate coil positioning may have influenced the reliability of the rapid mapping data. However, we note that exclusion due to coil angle has previously been found to occur in a small percentage of trials (3.3%) [14]. Moreover, the easy-to-maintain coil orientation of 90° to the anterior-posterior line along with the large amount of TMS mapping experience of the experimenters in our study is likely to contribute to even fewer errors. Nonetheless, we advise future studies to assess rapid mapping of masseter and quadriceps muscles accounting for incorrect orientation and angles. Another consideration is coil orientation. Whereas the present study induced a lateral-medial current for all muscles, some studies oriented the coil that generated anterior-posterior direction currents for mapping the quadriceps considering that this coil orientation could stimulate the corticospinal pathway to the lower limb muscles more efficiently [29]. However, it should be noted that this rationale is based on studies in the lower leg and foot muscles [62] and other studies demonstrated that the coil should be oriented to generate medial-lateral currents for optimal stimulation for the lower leg muscle [63; 64]. As altering coil orientation can influence the motor threshold and stimulate different populations of neurons [63; 64], further investigation is needed to examine the optimal coil orientation for mapping M1 corticomotor representations of the quadriceps muscles. Lastly, the study did not assess reliability and validity using different ISIs, as has been done previously [13]. Future studies are encouraged to re-assess the reliability and validity of rapid mapping of the knee/masseter muscles with different ISIs, as the optimal ISI may differ for different muscles.

## Conclusions

The process of acquiring TMS-evoked responses from the masseter and quadriceps muscles can be relatively challenging, as MEPs are typically collected while these muscles are under active contraction, which can lead to fatigue, especially during lengthy mapping sessions. Establishing methodologies that reduce map acquisition time for these muscles may improve the quality and participant tolerability of future experiments using masseter or quadriceps mapping. Here, we showed that maps produced using the rapid mapping method had modest absolute reliability, equivalence with the traditional mapping method for most outcomes/muscles, good to excellent relative reliability for map volume, but mixed relative reliability for other measures. Thus while rapid TMS mapping could be a promising substitute for traditional mapping of the non-upper limb muscles, further work is required to refine this methodology.

### Electronic supplementary material

Below is the link to the electronic supplementary material.

Supplementary Material 1

## Data Availability

The data that support the findings of this study are available from the corresponding author, NC, upon reasonable request.
