# Supplementary material for: The reliability and validity of rapid transcranial magnetic stimulation mapping for muscles under active contraction
Source: BMC Neurosci. 2024 Aug 30;25:43. doi: 10.1186/s12868-024-00885-w (PMC11363547; doi:10.1186/s12868-024-00885-w)
Supplement: Supplementary file 1 — Supplementary Material 1 [file 12868_2024_885_MOESM1_ESM.docx]

**The reliability and validity of rapid transcranial magnetic stimulation mapping for muscles under active contraction: Supplementary Material**


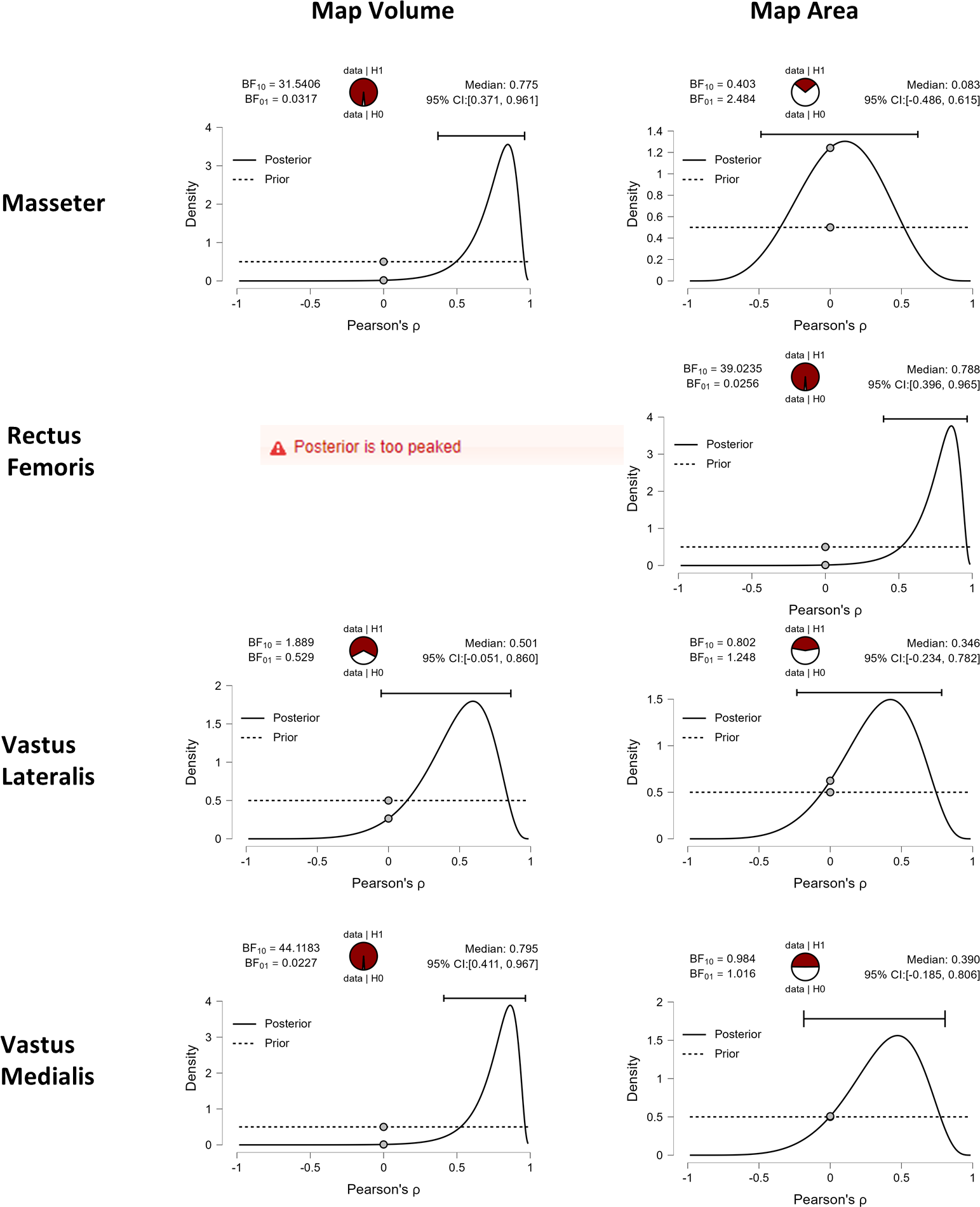


*Supplementary Figure 1.* Posterior distributions for the correlation in map volume and area between rapid mapping session 1 and session 2


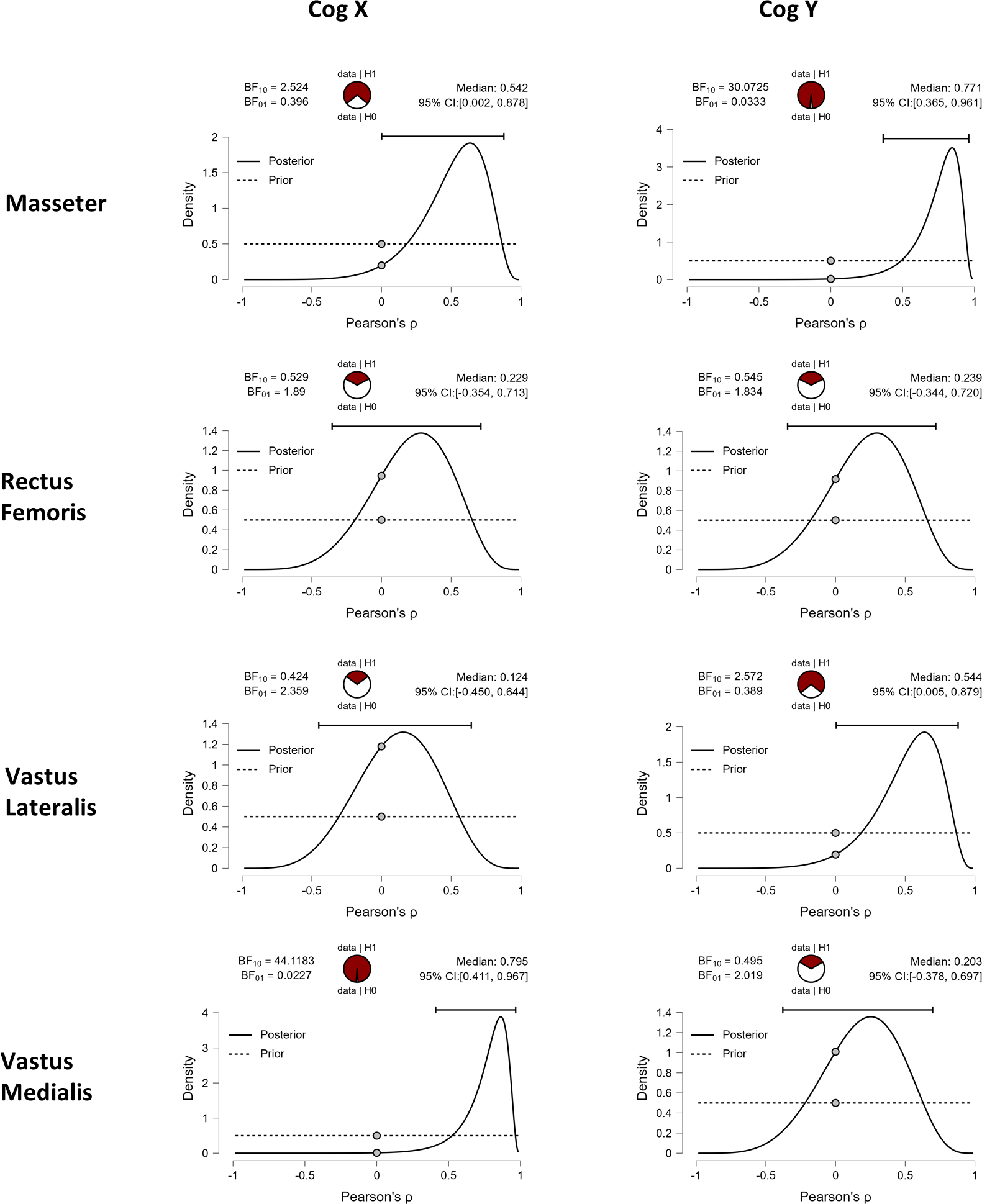


*Supplementary Figure 2.* Posterior distributions for the correlation in CogX and CogY between rapid mapping session 1 and session 2
